# Radium and Cancer

**Published:** 1914-05

**Authors:** R. H. L. Bibb


### EDITORIAL DEPARTMENT.

DR. F. E. DANIEL, Editor DR. R. H. L. BIBB, Associate Editor

DEPARTMENTS { EUGENICS: Drs. M. Duggan and T. Y. Hull.

WOMAN'S DEPARTMENT: Mrs. F. E. Daniel.

### Radium and Cancer.

THE NATURE OF CANCER.—Its exact pathology is unknown. Radium is one of the newest, most recent chemical elements with which physicians have had to deal. It has been studied by comparatively few, and its action and at times its nonaction, is poorly understood. Nevertheless, a brief retrospect of what has been learned of it and accomplished by it, may not be out of place.

Whilst radium will perhaps never be a "cancer cure," experience with it thus far furnishes grounds for hopes that when the profession is better informed as to the *real nature* of cancer, and as to the actual properties of radium and its action and how to control it, a great many forms of cancer which are today either not affected by it or, are made worse by its employment, will be greatly lessened if subjected to its influences whilst the disease may yet be curable, i. e., if treated with radium in time.

It is claimed, by the knowing ones, that the therapeutic action of radium on cancer cells is *selective*—i. e., its emanations have a *"selective affinity"* for the cells of cancer, which *is not caustic.* On healthy animal cells the power of radium is very pronounced, but not uniform. It can augment or diminish cell multiplication and can cause their death *en masse;* and whilst its influence on animal cells in general is marked, it is most pronounced upon the cells composing cancer, mut not equally so in all varieties of cancer—i. e., the cells of sarcoma are more influenced by it than are the cells of carcinoma, and the softer cancers than the harder ones. A scirrhus of the breast has been known to melt away under its influence, whilst another, in nowise distinguishable from the former, treated with the same tube of radium and in the same manner without appreciable benefit. Cancer of mucous membranes are less influenced by radium than are skin cancers; and some cancers, of every variety, are made worse by its use.

In some cancers the influence of radium is limited to the tissues in *immediate contact* with the capsule containing the substance, whilst in others of the same variety, histologically identical, whilst not extending throughout the entire tumor, is greatly more extensive, beneficially or injuriously as the case may be.

These are some of the as yet not explained "freaks" of radium which have to be understood an dexplained before the profession can use the substance intelligibly, or forcecast with any certainty what will be its effects in any given case of cancer of any variety of the many with which the profession is dealing daily.

It must not be forgotten that radium is as powerful for harm as it is for good—that it is "a two-edged sword"—even in the hands of those best qualified to use it. It may cure a case of cancer —and, seemingly it has cured many—and yet leave the patient in a condition of greater and longer suffering than before treatment, as has followed its use in cancer of the rectum and of the uterus. Sloughing of adjacent tissues is always a menace wherever it is applied; and has been known to extend to nearby arteries, to the stomach and intestines, and thus causing the death of the patient. Metastases of the malignant disease to healthy portions of the body has followed its use, as has thrombosis— the latter causing death of the victim within forty-eight hours. In all cases its use is followed by a greater or less systemic reaction—occasionally reaching the danger line. And the dangers that threaten the physician who handles radium should never be overlooked or underestimated; for they are as real and as many as are those attending use of the X-ray.

Thanks to its high price, radium, so far, has been beyond the reach of quacks and mountebanks, which fact has, doubtless, saved thousands of human lives and as much suffering; and, if its high price will only continue until competent and conscientious medical men and scientists can determine how to control it, when, where and upon whom to use it, it may be confidently hoped that the therapeutic value of radium in treating in cancer will be greatly extended and that the physician will be able to apply it only where its action is known, and where it may do the greatest good with the minimum possibility of harm—although it may never become "a cancer cure."—*R. H. L. Bibb.*

